# Do urban air pollutants induce changes in the thallus anatomy and affect the photosynthetic efficiency of the nitrophilous lichen *Physcia adscendens*?

**DOI:** 10.1007/s11356-023-30194-4

**Published:** 2023-10-13

**Authors:** Jakub Styburski, Kaja Skubała

**Affiliations:** Institute of Botany, Faculty of Biology, Jagiellonian University, Gronostajowa 3, 30-387 Kraków, Poland

**Keywords:** Chlorophyll fluorescence, Urban pollution, PSII maximal quantum yield, Nitrogen excess, Lichen anatomy, Particulate matter

## Abstract

Lichens are symbiotic organisms that are generally sensitive to air pollution due to their specific biological and physiological features. *Physcia adscendens* is a nitrophilous lichen well-known for being resistant to air pollution associated with progressive anthropopressure. The aim of this study was to investigate the effect of nitrogen oxides and suspended particulate matter (PM10 and PM2.5) on anatomical structure of the thallus and photobiont’s photosynthetic efficiency in *P. adscendens* inhabiting sites that differ in terms of air pollution level and thereby to determine the relevance of these pollutants for shaping the structure of the thallus and the physiological condition of the photosynthetic partner. We found that *P. adscendens* from polluted sites had increased thickness of the algal layer and the larger size of the algae cells, but a much lower ratio of the algal layer to the whole thallus. Lichens from highly polluted sites had also higher photosynthetic efficiency, which indicates a relatively good physiological condition of the photobiont. This indicates that the photobiont of *P. adscendens* is well-adapted to function under air pollution stress which may contribute to its success in colonizing polluted sites. Both changes in the anatomy of the lichen thallus and the efficiency of photosynthesis may be related to the enrichment of the environment with nitrogen. The increased photosynthetic efficiency as well as investment in the size of photobiont cells and growth mycobiont hyphae confirms that *P. adscendens* is well-adapted to urban conditions; however, the mechanism behind those adaptations needs more focus in the context of global environmental changes.

**Supplementary Information:**

The online version contains supplementary material available at 10.1007/s11356-023-30194-4.

## Introduction

Lichens are symbiotic associations basically composed of fungi (mycobiont) that are mainly representatives of *Ascomycota*, and algae/cyanobacteria (photobiont) belonging to *Chlorophyta* and/or *Cyanophyta.* However, recent studies indicate that this symbiotic association is more complex, and lichens should be rather treated as holobionts consisting of or coexisting with many associated microorganisms (Aschenbrenner et al. 2016; Hawksworth and Grube 2020). Due to the lack of any protecting layers or absorptive organs, lichens absorb both necessary nutrients and toxic elements through the whole surface of the thalli (Tyler 1989; Nash III 2008), which contribute to their great sensitivity to atmospheric purity changes (Hauck 2010) and makes them perfect biomonitors of air pollutants.

Nowadays, air pollution poses a big problem in urban agglomerations. Public transport, power plants, and industry are the main sources of air pollutants in urban areas (Mayer 1999; Cohen et al. 2004). According to a report of the European Environment Agency from 2022 (EEA 2022), despite a reduction in emissions in 2020, concentrations of particulate matter (including both PM2.5 and PM10), ozone (O_3_), and nitrogen dioxide (NO_2_) regularly exceed the European Union air quality standards. Nitrogen pollution is commonly considered one of the major environmental concerns related to anthropogenic activities in the last decades (Galloway et al. 1995; Erisman et al. 2007). Nitrogen oxides pollution in cities comes mainly from traffic due to the widespread use of vehicles in urban centres (McDuffie et al. 2020). Thus, urban centres are important point sources of nitrogen pollution (Pataki et al. 2006). Particulate matters PM10 and PM2.5 are the second pollutant of major concern. They consist of a variety of components, such as organic compounds, metals, acids, soil, and dust, of which many are hazardous (US Environmental Protection Agency 1996). They are emitted mainly by the combustion of fuels for domestic heating, industrial activities, agriculture, and road transport.

Particulate matter can cause both acute and chronic injuries in plants and lichens. Nevertheless, most research is concerned with plants, and it has been shown that exposure to high level of PM in the atmosphere can cause leaf injury, stomatal damage, decreased photosynthetic activity, disturbance in membrane permeability, and changes in nitrate accumulation ability and amino acid levels (Pavlík et al. 2012; Rai 2016; Popek et al. 2018). With regard to lichens, many injury symptoms have been reported due to exposure to various components of particulate matter. For example, polycyclic aromatic hydrocarbons (PAHs) caused oxidative stress to lichen mycobiont (Lucadamo et al. 2021). Exposure to PM is also especially harmful to lichens due to heavy metals. Excessive amounts of heavy metals that are accumulated in lichen thallus can cause a variety of injury symptoms, such as changes in cell membrane integrity, membrane lipid peroxidation, ultrastructure disorders, photosynthesis impairment, or chlorophyll degradation (e.g. Cuny et al. 2004; Sanità di Toppi et al. 2005; Kováčik et al. 2018; Osyczka and Rola 2019).

In general, nitrogen is an essential nutrient for formation of proteins and nucleic acids. However, its excess in the environment causes negative effects on various living organisms. The effect of nitrogen compounds on lichen physiology is widely studied, and various research showed that both nitrate (NO_3_^−^) and ammonium (NH_4_^+^) forms of nitrogen can cause significant changes in physiology of lichens, for example, induce cell membrane damage and membrane lipid peroxidation and cause a decrease in photosynthetic efficiency as well as chlorophyll a and ergosterol contents (e.g. Gaio-Oliveira et al. 2004; Munzi et al. 2009, 2010; Paoli et al. 2010, 2015). The effect of excess nitrogen on lichen vitality depends on lichen species and the form of nitrogen. For example, Munzi et al. (2012) revealed that increased concentrations of ammonium chloride do not affect the cell membrane integrity; however, other nitrate and ammonium salts do (Munzi et al. 2009). However, lichens take up mainly ammonium rather than nitrate forms (Palmqvist and Dahlman 2006), since nitrates need to be reduced to ammonium, which entails additional energy costs (Hauck 2010). Ammonium in gaseous form has been shown to directly affect photosynthetic apparatus by decreasing *F*_V_/*F*_M_ parameter (Paoli et al. 2010). Nevertheless, the response of lichens to nitrogen pollution is a complex phenomenon. Munzi et al. (2010) have noticed that it depends on species, time of exposition, and dose of compound. On the other hand, nitrogen, being a strongly nutritious element, could also have a positive impact on lichen thalli. The ammonium ions caused the concentration of chlorophyll a and b to increase, as a result of carbon assimilation capability, through increased investment of lichen thalli in photobiont cells (Palmqvist and Dahlman 2006).

Air pollutants besides affecting the physiology of the lichen thallus may also change thallus morphology and anatomy. Apart from the first visible signs of lichen response to pollution, such as convolution, bleaching, changes in the thallus colour, or chlorotic and necrotic patches (Goyal and Seaward 1982; Otnyukova 2007), the changes of external morphology, growth abnormalities, ultrastructural modifications, and accumulation of calcium oxalate crystals were found in various lichen species exposed to different pollutants (e.g. Mikhailova 2007; Paoli et al. 2015; Osyczka et al. 2018).

The main aim of this study was to compare the anatomical characteristics of the thallus and the photosynthetic performance of photobiont in nitrophilous lichen *Physcia adscendens* (Fr.) inhabiting sites that differ in terms of air pollution with nitrogen oxides and suspended particulate matter (PM 10 and PM2.5), and thereby to determine the relevance of these factors for shaping the structure of the thallus and the physiological condition of the photosynthetic partner.

## Materials and methods

### Target lichen species

*Physcia adscendens* H. Olivier is a foliose lichen with whitish to very pale grey narrow-lobed thalli often with dark-tipped marginal cilia and helmet-shaped soralia (Nimis and Martellos 2022). This is a widespread holarctic lichen that readily colonizes habitats enriched with nitrogen compounds; thus, it is regarded as an indicator of high nitrogen levels in the environment with an indicator value of 6 according to Wirth (2010). Moreover, *P. adscendens* is considered as air pollution–resistant species (Gilbert 1973).

### Study sites and lichen sampling

The study sites were selected based on the location of air pollution measuring stations of the Regional Department of Environmental Monitoring in Kraków, which is a government institution established to control compliance with environmental protection regulations as well as research and assessment of the state of the environment. Four study sites located within 100 m from the following air measuring stations were selected: Kraków, Aleja Krasińskiego avenue (code PL0012A; A-KRK); Kraków, Dietla street (code PL0641A; D-KRK); Kraków, Bulwarowa street (code PL0039A; NH-KRK); and one near city where air pollution is far less than in Kraków, Trzebinia town (code PL0552A; T). The measuring stations have been selected to differ in the level of air pollution. At each study site, twenty lichen samples (individuals) were collected from five trees. The selected trees represented *Acer* spp. and had a diameter at breast height (DBH) of 18–25 cm, which corresponds to the age of the tree 30–40 years. All trees were solitary trees, loosely dispersed near the communication routes, which provided relatively similar microclimatic conditions at each site. Details on basic climatic parameters in the study area are provided in Table S2 and S3 in the “Supplementary information.” Lichen thalli were collected from the bark using tweezers and a penknife. They were collected from each cardinal side of the trunks at 120–160 cm above the ground to make a composite sample. The samples were collected between July and September of 2018. For the measurements of the photosynthetic activity of the photobiont, the material from the same study sites and trees was collected using similar methods. Samples were packed in paper bags and transported to the lab. In the laboratory, all foreign materials adhering to the surfaces of the thalli, e.g. bark, moss, and soil particles, were carefully removed. In addition, lichen thalli were gently rinsed once with deionized water to remove loose dust particles. Prior to chlorophyll fluorescence measurements, lichen samples were hydrated and acclimatized in the laboratory at 20 °C and 95% relative humidity in a climatic chamber.

### Air pollution data

In Kraków (S Poland), particulate matter (2.5 and 10 μm) and NO_x_, including NO_2_ and NO, were classified as the most problematic air pollutants. According to report of The National Centre of Emissions Management from 2017, they originate mainly from combustion process (in industry and households) and road traffic, respectively (KOBiZE 2017). The geographic location of Kraków prevents air pollution from being cleared out, forcing lichens and plants to deal with it all year. The air pollution data were derived from the Chief Inspectorate of Environmental Protection in Poland. Data concerning four air pollutants were taken into account: NO, NO_2_, NO_x_, PM2.5, and PM10. Characteristics of the level of air pollutants at individual study sites for the period from January 2016 to May 2018 are presented in Table 1. In our study, nitrogen oxides and PM are used as a proxy of the level of pollution; however, the influence of other factors has not been taken into account, and therefore, their influence on observed results cannot be excluded.

**Table 1:** Mean values ± SD (μg/m^3^) of individual air pollutants at different air pollution measuring stations averaged over a 17-month period (January 2016 to May 2018). The raw data were derived from Chief Inspectorate of Environmental Protection, Poland

	Trzebinia (T)	Bulwarowa (NH-KRK)	Dietla (D-KRK)	Aleja Krasińskiego (A-KRK)
NO_2_	17.9 ± 7.2	29.0 ± 5.2	42.7 ± 6.3	60.3 ± 5.9
NO_x_	23.3 ± 10.5	60.1 ± 23.0	98.6 ± 24.9	199.2 ± 36.2
NO	3.5 ± 2.4	20.3 ± 12.1	36.5 ± 14.0	90.7 ± 23.4
PM10	36.4 ± 17.0	43.3 ± 21.0	50.1 ± 22.6	57.8 ± 27.1
PM2.5	-	29.8 ± 17.2	-	40.0 ± 21.6

### Anatomical measurements

Precise cross-sections of lichen thalli were done using a microtome (Microm, Adams Instrumenten) and stained with a lactophenol blue solution. Anatomical observations were made under light microscope (Zeiss Axioskop 2 Plus, Carl Zeiss) using magnifications 20× (for measurements of thallus layers) and 36× (for measurements of anatomical features of photobiont cells). The photos were taken with camera Moticam 1080 (Motic, Hong Kong, China). Two hundred photos of thallus cross-sections were taken for each study site. The measurements were made using Motic Images Plus 2.0. The thickness of the upper and lower cortex layers, the algal layer, the whole thallus, the surface area of the algal cell, the number of algal cells per 1000 μm^2^ of the algal layer, and the density of the algal cells were measured. To assess the density of algal cells within algal layer, the following formula was used:

$$D=\frac{\sum S}{s}\times 100\%$$

where:

D: density of algal cells
∑*S*: algebraic sum of all algal cell surface areas in measured surface
s: surface area on which algal cells were measured

The number of measurements for individual anatomical features in lichen thalli collected from particular study sites is shown in Table 2.

**Table 2:** Number of measurements of anatomical characteristics in lichens collected from different study sites. Next to anatomical features, measurement units are given in parentheses.

Anatomical feature	Study site				
	Trzebinia (T)	Bulwarowa (NH-KRK)	Dietla (D-KRK)	Aleja Krasińskiego (A-KRK)	All
Upper cortex layer thickness (μm)	1800	1920	1700	1360	6780
Algal layer thickness (μm)	1800	1920	1700	1360	6780
Lower cortex layer thickness (μm)	1780	1920	1680	1360	6740
Whole thallus thickness (μm)	1780	1920	1680	1360	6740
Algal layer thickness/whole thallus thickness	1780	1920	1680	1360	6740
Surface area of algal cell (μm^2^)	1831	1359	1282	983	5455
Number of algal cells per 1000 μm^2^	84	77	71	59	291
Density of algal cells	84	77	71	59	291

### Chlorophyll fluorescence measurements

Chlorophyll fluorescence measurements were performed using Handy-PEA+ fluorimeter (Plant Efficiency Analyzer, Hansatech Instruments Ltd, Norfolk, England). The samples were dark-adapted for 25 min before measurements. Chlorophyll fluorescence was induced by red light (wavelength 650 nm). Data were recorded after a saturating light pulse of 2400 μmol m^−2^ s^−1^. Fluorescence emission was recorded with a time span from 10 μs to 1 s.

The vitality of the lichen photobiont was assessed by the maximum PSII quantum yield in dark-adapted state as inferred from Chl *a* fluorescence emission: *F*_V_/*F*_M_ = (*F*_M_ – *F*_0_)/*F*_M_, where *F*_0_ and *F*_M_ are minimum and maximum Chl *a* fluorescence and *F*_V_ = (*F*_M_ – *F*_0_) is the variable fluorescence. The *F*_0_ parameter constituted the value calculated using extrapolation back to time point 0 using least squares regression of the first few data points. The second selected parameter was the performance index (PI_ABS_), which evaluates the overall photosynthetic performance (Strasser et al. 2004). For a more precise analysis of photosynthetic apparatus conditions, other chlorophyll fluorescence parameters were also analysed (see Table S1).

### Data analysis

One-way ANOVAs (*p* < 0.05) followed by Tukey’s HSD post hoc tests were used to verify significant differences in concentrations of air pollutants between study sites and in lichen anatomical features as well as the *F*_V_/*F*_M_ and PI_ABS_ parameters between lichens collected from particular study sites. Before the analyses were performed, the normality distribution in particular groups was checked using the Kolmogorov-Smirnov test. The Brown-Forsythe tests were used to verify the homogeneity of variances.

The spider plots were created to illustrate the changes of various parameters characterizing PSII functionality for lichens collected at particular study. The graphs were used to verify if there are more sensitive parameters than *F*_V_/*F*_M_ that could indicate the effect of stress. The plots were based on the values normalized to the T site (least polluted site).

## Results

### Differences in air pollution between study sites

**Fig. 1:**
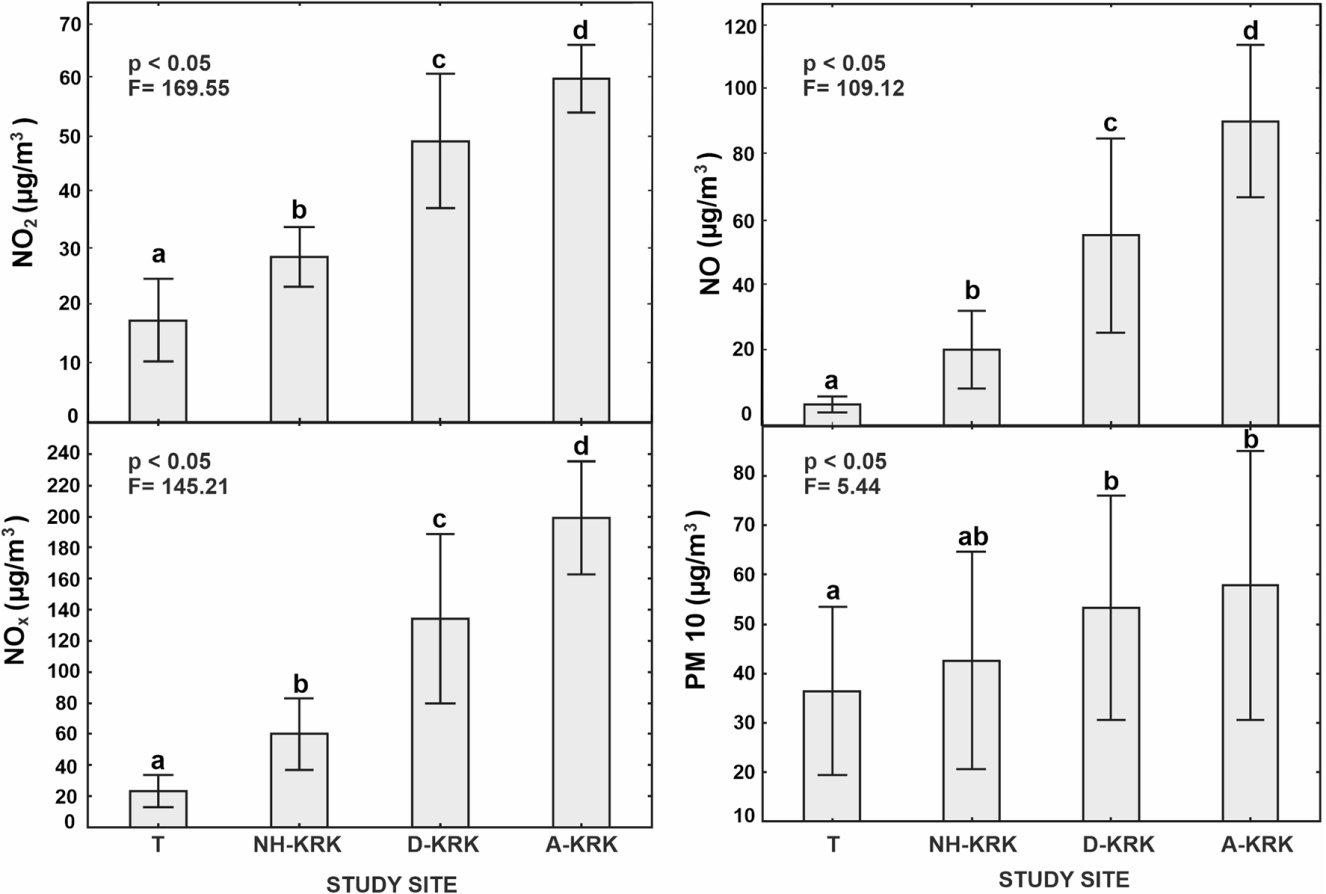
Mean (±SD) of NO_2_, NO, NO_x_, and PM10 concentrations in the air at different air pollution measuring stations located in the vicinity of study sites; calculated for the period from January 2016 to May 2018. The results of one-way ANOVA are provided. The different letters above the bars indicate statistically significant differences (*p* < 0.05)

The mean concentrations of air pollutants in the period of 2.5 years before lichen collection differed significantly between study sites (one-way ANOVA; *p* < 0.05; Fig. 1). The highest concentrations of NO, NO_x_, NO_2_, and PM_10_ were recorded on the A-KRK site followed by D-KRK and NH-KRK and achieved the lowest values at the T site. Regarding the concentrations of nitrogen oxides, each site differed significantly between each other. In the case of PM10 concentration, A-KRK and D-KRK sites differed significantly only from the T site, while the NH-KRK site did not differ significantly from the remaining sites.

### Differences in thallus layer thickness

**Fig. 2:**
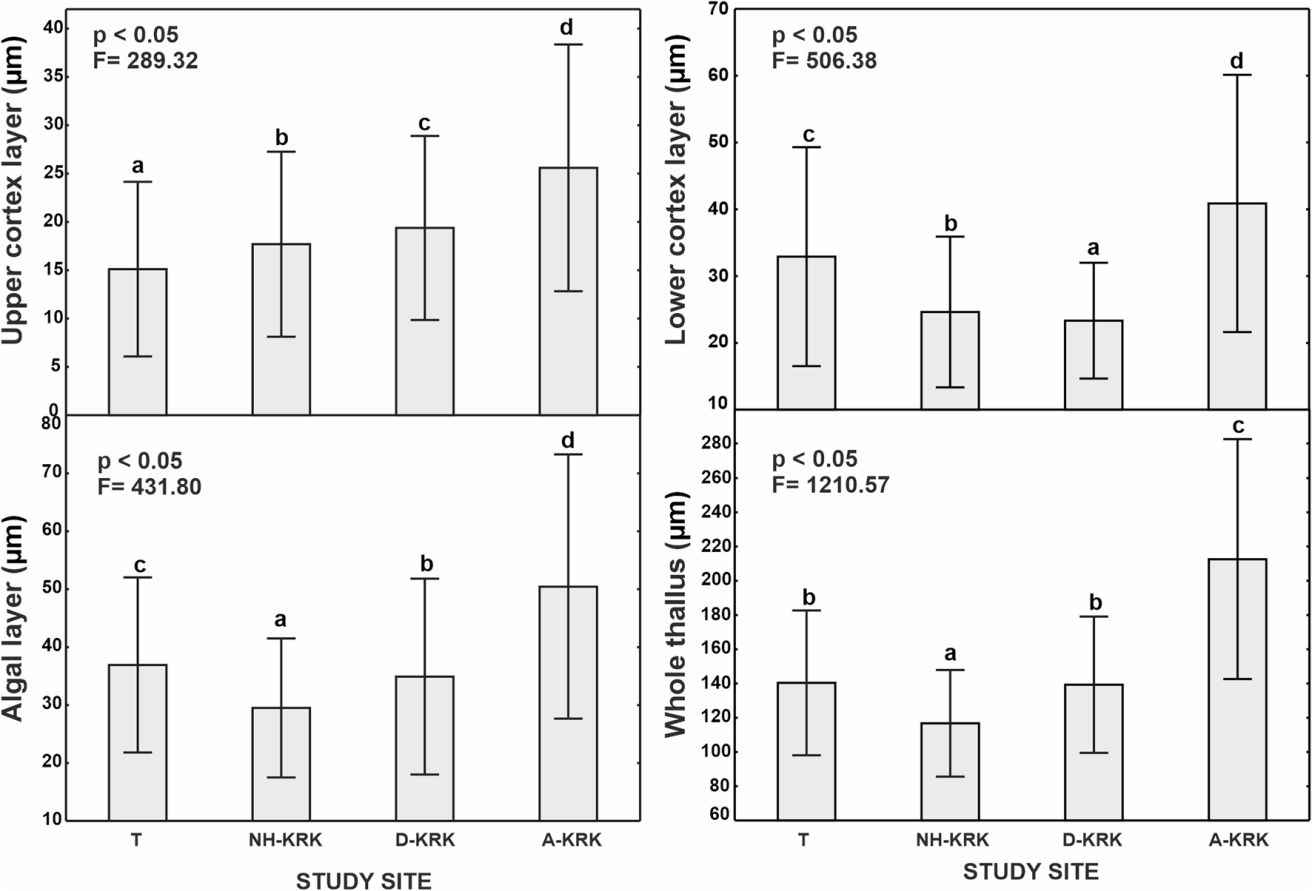
Mean (±SD) of the thickness of the layers of *P. adscendens* thalli collected at particular study sites. The results of one-way ANOVA are provided. The different letters above the bars indicate statistically significant differences (*p* < 0.05)

**Fig. 3:**
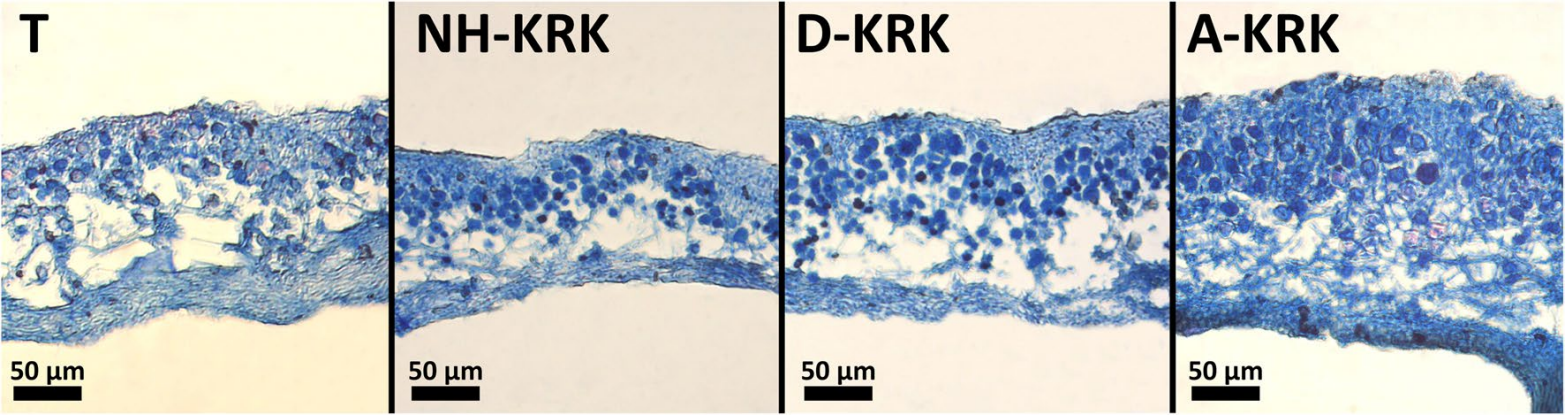
Exemplary cross-sections of *P. adscendens* thalli collected from particular study sites

The thickness of each of the analysed thallus layers differed significantly between study sites (one-way ANOVA; *p* < 0.05; Figs. 2 and 3). The upper cortex layer thickness increased in line with the increase in air pollutant concentrations, being significantly the highest at the A-KRK site and the lowest at the T site. Another pattern was found for the lower cortex layer, the algal layer, and the whole thallus thickness. Regarding algal layer thickness and the whole thallus thickness, the lowest values were recorded at the NH-KRK site, whereas the A-KRK site was characterized by significantly the highest values of these parameters. Lichens collected at D-KRK and T sites had intermediate values of these parameters, whereby the algal layer thickness was significantly higher at the T site, while no significant differences in the whole thallus thickness between T and D-KRK sites were found.

### Differences in algal anatomical features

**Fig. 4:**
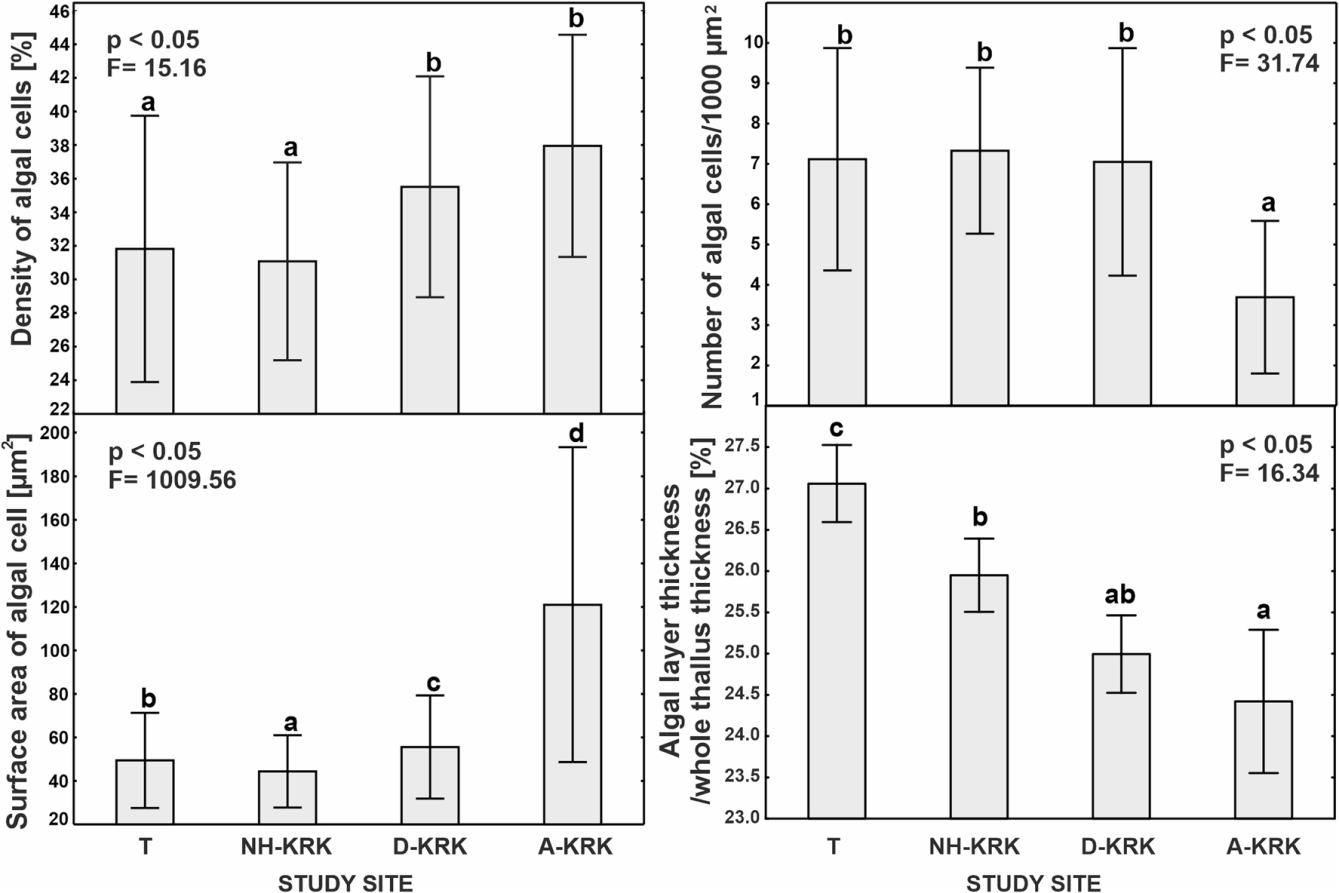
Mean (±SD) of algal anatomical parameters measured in *P. adscendens* thalli collected at particular study sites. The results of one-way ANOVA are provided. The different letters above the bars indicate statistically significant differences (*p* < 0.05)

The algal anatomical parameters differed significantly between study sites (one-way ANOVA; *p* < 0.05; Fig. 4). The surface area of photobiont cells was the highest at the A-KRK site, where also the highest concentrations of air pollutants occur. The values of this parameter at the A-KRK site were approximately twice that of NH-KRK, D-KRK, and T sites. Significantly the lowest surface area of photobiont cells was recorded at the NH-KRK site. The lowest number of algal cells per 1000 μm^2^ was recorded at the A-KRK site, and it differed significantly from the remaining study sites where approximately 7 algal cells were present on the surface of 1000 μm^2^. Regarding the density of algal cells, two groups of sites can be distinguished, the T and NH-KRK sites with significantly lower values of this parameter and D-KRK and A-KRK sites with significantly higher density of algal cells. The study sites within these two groups did not differ significantly from each other.

### Photosynthetic efficiency

**Fig. 5:**
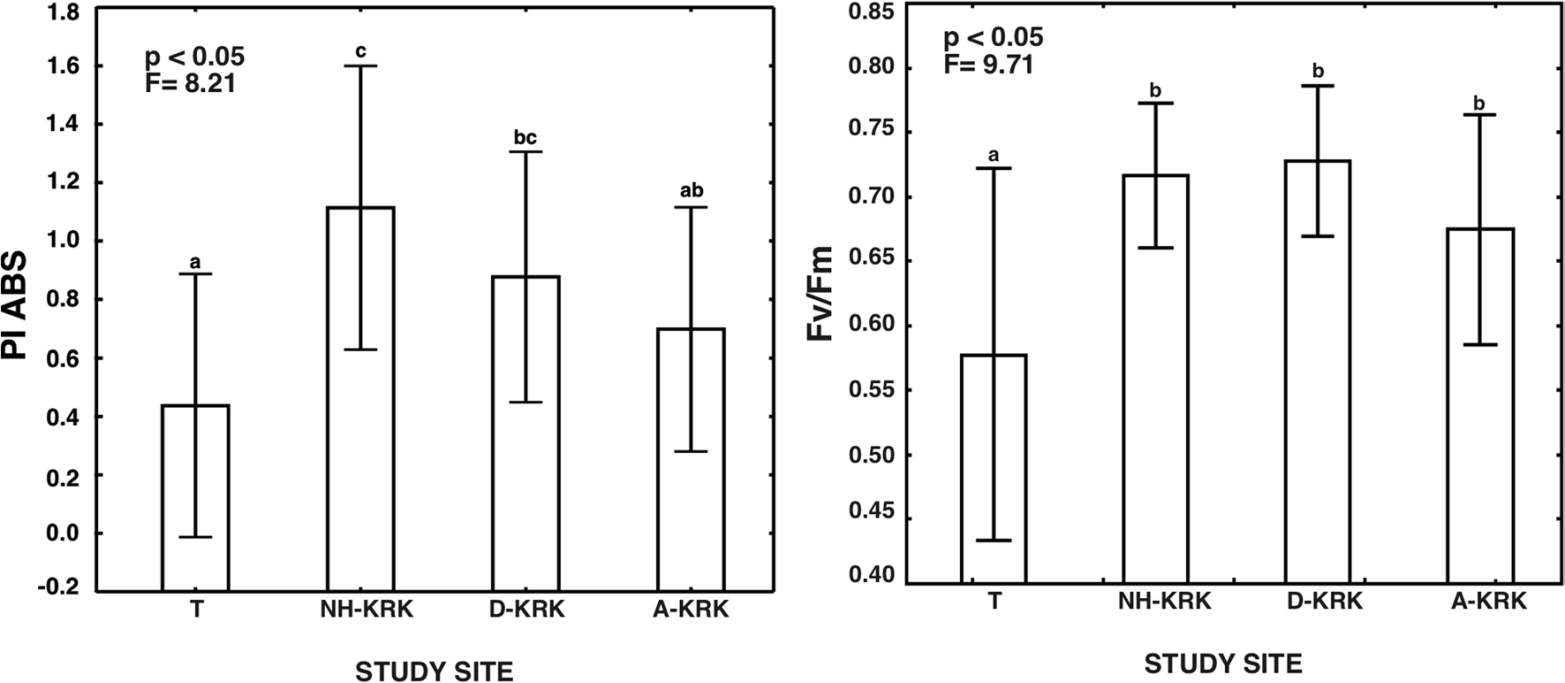
Mean (±SD) of PI_ABS_ and *F*_V_/*F*_M_ in *P. adscendens* thalli collected at particular study sites. The results of one-way ANOVA are provided. The different letters above the bars indicate statistically significant differences (*p* < 0.05)

Both the maximum quantum yield of PSII photochemistry (*F*_V_/*F*_M_) and PI_ABS_ differed significantly between study sites (one-way ANOVA; *p* < 0.05; Fig. 5). The mean value of *F*_V_/*F*_M_ in lichen thalli from the T site was the lowest and differed significantly from the remaining sites. Lichens collected from three sites located in Kraków did not differ significantly from each other and the values of *F*_V_/*F*_M_ oscillated around 0.65–0.75. Regarding PI_ABS_, lichens from NH-KRK and D-KRK sites located in Kraków had significantly higher values of this parameter compared to the least polluted site (T).

**Fig. 6:**
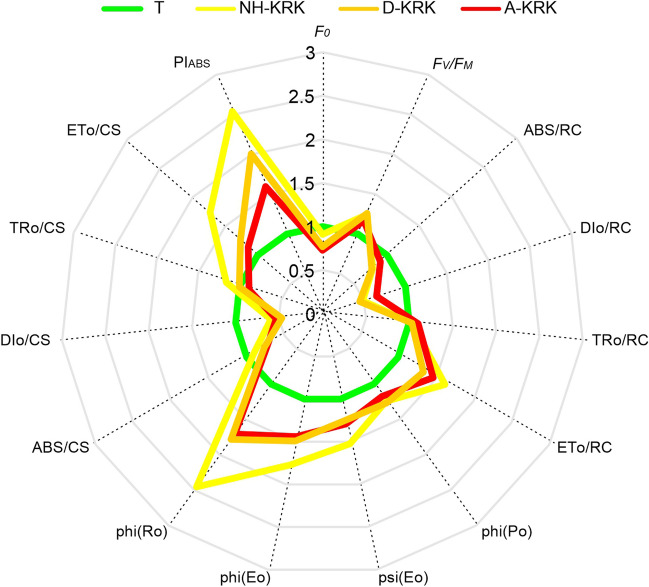
The spider plots showing the differences in various photosynthetic parameters characterizing PSII functionality in *P. adscendens* thalli collected at particular study sites. The plots are based on normalized values to the least polluted site (T), enabling comparison of the variables measured on different scales. For a detailed description of selected chlorophyll fluorescence (OJIP) parameters, see Table S1

Regarding various fluorescence parameters, the performance index (potential) for energy conservation from photons absorbed by PSII to the reduction of intersystem electron acceptors (PI_ABS_), quantum yield of reduction of end electron acceptors at the PS I acceptor side (phi (R_0_)), probability that a trapped exciton moves an electron into the electron transport chain beyond QA (psi (E_0_)), electron transport flux per excited cross-section (ET_0_/CS), and reaction centre (ET_0_/RC) showed the greatest differences between study sites. As a rule, lichens collected from the sites located in Kraków City were characterized by the highest values of these parameters (Fig. 6).

## Discussion

Despite a great deal of research being done, the effect of air pollution on lichen morphology/anatomy is still poorly recognized. Atmospheric pollutants can affect growth and cause visible changes in the morphology and anatomical structure of the lichen thallus (e.g. Bennett 2002; Wakefield and Bhattacharjee 2012). Consequently, such changes can potentially be used as indicators for assessment of air pollution level (Nimis et al. 2002). For example, Bennett (2002) found that the algal layer ratio and the total thallus thickness are useful indicators of pollution in *Parmelia sulcata*. Our results showed that some anatomical features clearly differ between thalli collected from sites with different levels of air pollution. Regarding the photobiont layer, Rola and Osyczka (2019) found that high pollution level induced higher ratios of the algal layer to the whole thallus, as well as higher thickness of algal layer. Similarly, Goyal and Seaward (1982) observed significantly greater relative algal layer thickness of *Peltigera* spp. collected at polluted sites compared to background sites. Our results confirmed this trend in *P. adscendens* that had considerably thicker algal layers at highly polluted sites. Contrarily, thalli of *Parmelia sulcata* had thinner algal layers in polluted areas, which may indicate poorer thallus vitality of this species under air pollutant exposure (Bennett 2002). The photobiont cells at the highly polluted site were twice as large as at the other sites (Fig. 4). However, quality does not go hand in hand with quantity since the number of the photobiont cells within 1000 μm^2^ of the algal layer was twice lower than at the other sites. Contrarily, Rola et al. (2021) found smaller algal cells and greater number of algal cells within the photobiont layer in *Cladonia rei* inhabiting heavy metal–polluted sites. This shows that different species may react differently to stress factors and could suggest that lichens invest in either photobiont cell quality (cell size) or quantity (cell number), but more research is needed to fully understand this phenomenon. Moreover, the observed trend may also be related to the fact that air pollutants are likely to supress algal cell division as was observed in *Hypogymnia physodes* exposed to emissions of a copper-smelting plant (Mikhailova 2007). The greater thickness of the algal layer observed in our study does not necessarily indicate better physiological condition of the photobiont since lichens collected at polluted sites had lower proportion of photobiont in relation to mycobiont compared to lichens collected from less polluted sites (Fig. 4).

The thalli of *P. adscendens* collected at the site characterized by the highest pollution level had also the thickest upper and lower cortex layer and the whole thallus. Similar observations were made by Goyal and Seaward (1982) in *Peltigera* spp. thalli from a site with polluted air compared to a background habitat. Our results are also in line with the report of Estrabou et al. (2004) who demonstrated in *Physcia endochrysea* that thickness of upper cortex layer was higher in lichens collected from urban area compared to rural site. The observed thicker cortical layers under air pollution could be associated with direct influence of excessive nitrogen supply, thereby increased carbon assimilation and as a result intensive growth of fungal hyphae (Palmqvist and Dahlman 2006). Nevertheless, the physiological and anatomical responses of lichens in environmental context are often complicated by the multivariate complex of environmental factors that act simultaneously in the natural habitat of lichens. Generally, in the absence of other stressors, light intensity, temperature, and moisture are key factors for lichen physiology (Nash III 2008; Stanton et al. 2023). Therefore, an additional influence of microclimatic factors and biotic interactions on the observed changes in the anatomy of lichens between sites cannot be excluded, although we tried to minimize microclimatic and microhabitat differences between the sites.

With regard to the efficiency of the photosynthetic process, the PI_ABS_ and *F*_V_/*F*_M_ parameters reached the highest values in lichens from polluted sites, while the values at the least polluted site were distinctly lower. This indicates a relatively good physiological condition of the photobiont under stress conditions. Perhaps the photosynthetic apparatus in lichens at sites with a low level of air pollution does not show much activity in terms of binding carbon dioxide, because it does not have to—in stress-free conditions, minimal involvement may be sufficient. Similar relationships were observed in epigeic lichens growing on soils polluted with heavy metals, where the essential photosynthetic process remains unaffected, despite the high accumulation level of toxic trace elements in lichen thalli (Rola et al. 2019). The global performance index (PI_ABS_) proved to be more responsive than *F*_V_/*F*_M_. Similar observations were made by Paoli et al. (2010); however, in this case, high atmospheric ammonia concentrations resulted in a decrease of this parameter in *Evernia prunastri* and *Pseudevernia furfuracea*. Moreover, the remaining OJIP-derived chlorophyll fluorescence parameters indicated the decreased efficiency of photosynthesis in the least polluted site. Consequently, our results indicate that photobiont of *P. adscendens* is well-adapted to function under air pollution stress, which may contribute to its success in colonizing polluted sites.

Both changes in lichen anatomy and the efficiency of photosynthesis may also be related to the enrichment of the environment with nitrogen. The concentration of nitrogen oxides at all air pollution measuring stations located in Kraków City exceeded the acceptable level according to Regulation of the Minister of the Environment (Dz.U. of 2008, No 47, item 281). Road traffic emissions release both nitrate and ammonia compounds (Gadsdon and Power 2009) and both forms can be utilized by lichens for their growth (Dahlman et al. 2004). Nitrogen enrichment may have different effects on lichen structure due to different physiological responses of fungal and algal partners to this nutrient (Dahlman 2003). Since the mycobiont constitutes most of the lichen biomass, it can be assumed that the mycobiont is mainly responsible for nitrogen acquisition and translocation within the thallus. Nevertheless, the results of fertilization experiments suggested that the effect of excess N on lichen growth may be biased towards photobiont growth rather than mycobiont growth (Roy-Arcand et al. 1989). This could explain the increased thickness of the algal layer and the larger size of the algae cells in lichens from polluted sites. Similarly, Kauppi (1980) observed an excessive growth of algae cells in the thallus of *Cladina* lichens from environment highly enriched with nitrogen. Although elevated nitrogen levels proved to have deleterious effects on the symbiotic equilibrium, with green algal photobionts benefiting disproportionately (Palmqvist et al. 2017; Gaio-Oliveira et al. 2005), the increased N availability may contribute to both increased photobiont proportions and photosynthetic rates in certain lichen species and also decreased mycobiont proportion in the thalli after nutrient treatments (Johansson et al. 2011). The low nitrate utilization capacity of fungi, while high in green algae (Avalos and Vicente 1985; Crittenden 1996), may suggest that the photobiont would make better use of the nitrogen source compared to the mycobiont (Hawkins et al. 2000; Grossman and Takahasi 2001). The study conducted by Dahlman et al. (2003) on *Hypogymnia physodes* and *Platismatia glauca* subjected to nitrogen fertilization suggested that the relative investment of N in the lichen thallus has been radically changed in favour of the photobiont and indicated that the increased photosynthesis efficiency may also be an adaptation to alleviate the carbon cost of high accumulation of N. This is in line with our results since greater photosynthetic efficiency in lichen thalli from the most polluted sites was observed. On the other hand, we found a much lower ratio of algal layer to the whole thallus in lichens from polluted sites, which could be associated with increased fungal hyphal growth in relation to the volume of the thallus occupied by algal cells. This may in turn suggest that the mycobionts must ensure sufficient nitrogen investment for the photobionts to achieve efficient photosynthesis in order to meet fungal accompanying carbohydrate needs. It has been revealed that in lichens allocation of N to the photobiont could result in increased chlorophyll concentration and photosynthetic capacity when the N load is within a tolerance threshold of a certain species (Palmqvist et al. 1998), but when the tolerance level is exceeded, the response could be reversed, and detrimental effects are likely to occur (Hauck 2010). Nevertheless, it should be borne in mind that response to N enrichment is not only species-specific, but also dependent on the thallus N concentrations, so further research focusing on ammonia and nitrate concentrations in the environment and nitrogen accumulation inside lichen thallus may provide further information helping to explain the changes we observed in the anatomy and physiology of *P. adscendens*.

## Conclusions

Lichens from highly polluted sites had higher photosynthetic efficiency, which indicates a relatively good physiological condition of the photobiont. The increased thickness of the algal layer and the entire thallus together with the large photobiont cells and the high efficiency of photosynthesis may indicate a high level of air pollution. Both changes in the anatomy of the lichen thallus and the efficiency of photosynthesis may be related to the enrichment of the environment with nitrogen. The increased photosynthetic efficiency as well as investment in the size of photobiont cells and growth mycobiont hyphae confirms that *P. adscendens* is well-adapted to urban conditions; however, the mechanism behind those adaptations needs more focus in context of global environmental changes. Our results may be important in the context of better understanding the functioning of nitrophilous lichens in urban conditions, which are characterized by increased levels of air pollutants.

### Supplementary information

ESM 1 (PDF 235 kb)

## Data Availability

All data supporting the findings of this study is included in this manuscript.
