# Supplementary material for: Do urban air pollutants induce changes in the thallus anatomy and affect the photosynthetic efficiency of the nitrophilous lichen Physcia adscendens?
Source: Environ Sci Pollut Res Int. 2023 Oct 13;30(52):112336–46. doi: 10.1007/s11356-023-30194-4 (PMC10643396; doi:10.1007/s11356-023-30194-4)
Supplement: Supplementary file 1 — (PDF 235 kb) [file 11356_2023_30194_MOESM1_ESM.pdf]

Supporting information (electronic supplementary materials available on-line)

Environmental Science and Pollution Research

Do urban air pollutants induce changes in the thallus anatomy and affect the photosynthetic efficiency of the nitrophilous lichen *Physcia adscendens*?

Jakub Styburski[1*], Kaja Skubała[1]

[1]Institute of Botany, Faculty of Biology, Jagiellonian University, Gronostajowa 3, 30-387 Kraków, Poland

*Corresponding author. E-mail address: jakub.styburski@doctoral.uj.edu.pl

Table S1. The selected chlorophyll fluorescence (OJIP) derived parameters calculated on the basis of fast fluorescence kinetics used in the analysis.

Fluorescence parameter	Description
Basic	
F_0	Minimal fluorescence intensity
F_V/F_M	Maximum quantum yield of PSII photochemistry
The specific energy fluxes per reaction centre (RC)	
ABS/RC	Specific absorption flux per reaction centre
DI_0/RC	Dissipated energy flux per reaction centre
TR_0/RC	Trapped energy flux per reaction centre
ET_0/RC	Electron transport flux per reaction centre
Quantum yields and efficiencies	

Phi (P_0)	Probability that an absorbed photon will be trapped by the reaction centre of PSII
Psi (E_0)	Probability that a trapped exciton moves an electron into the electron transport chain beyond QA
Phi (E_0)	Quantum yield of electron transport
Phi (R_0)	Quantum yield of reduction of end electron acceptors at the PS I acceptor side
Phenomenological energy fluxes per excited cross-section (CS)	
ABS/CS	Specific absorption flux per excited cross-section
DI_0/CS	Dissipated energy flux per excited cross-section
TR_0/CS	Trapped energy flux per excited cross-section
ET_0/CS	Electron transport flux per excited cross-section
Performance index	
PI_{ABS}	Performance index (potential) for energy conservation from photons absorbed by PSII to the reduction of intersystem electron acceptors

Table S2. The selected climatic parameters measured between January 2016 and December 2018 at two different meteorological stations in the study area (IMGW code: 250190390 and 350190566). Data source: The Institute of Meteorology and Water Management - National Research Institute 2023.

	KRAKÓW-BALICE				KRAKÓW-OBSERWATORIUM			
**Month of the year**	**Average maximum temperature [°C]**	**Average monthly temperature [°C]**	**Precipitation [mm]**	**Average relative humidity [%]**	**Average maximum temperature [°C]**	**Average monthly temperature [°C]**	**Precipitation [mm]**	**Average relative humidity [%]**
**I**								
2016	13.4	-2.3	19.4	86.6	15.5	-1.6	26.4	81.6
2017	5.7	-5.7	7.2	83.5	6.9	-4.5	9.6	78.4
2018	10.7	0.9	17.6	84.7	12.3	1.7	22.1	82.7
**II**								
2016	14.9	4	85.5	83.9	16.1	4.6	104.9	80.5
2017	16.6	0.1	31	83.3	17.2	1	36.6	77.9
2018	8.1	-3	9.8	85	9.2	-2.3	13.6	80
**III**								
2016	17.1	4.7	17.9	79.2	18.5	5.4	30.8	75.5
2017	20.4	6.2	38.2	75.1	21.9	6.9	41.6	72.4
2018	17.2	0.6	19.4	76.8	18.8	1.3	23.9	73.1
**IV**								
2016	26	9.2	51.4	74.3	27.3	10.2	66	70.5
2017	24.1	7.5	92	76	25.8	8.4	115.7	71.1

2018	29.2	13.8	9.7	62.9	30	14.5	10.2	58.3
**V**								
2016	27.7	14.2	45.1	73.3	28.7	15.1	56.7	69.5
2017	29.9	13.7	52.9	78.3	30.7	14.7	64.5	72.1
2018	30.5	17.1	68.6	67	30.3	17.7	43.2	63.4
**VI**								
2016	33.6	18.7	57.3	68.9	35.3	19.7	46.7	64.5
2017	33.2	18.8	30	65.3	33.9	19.9	46.9	58.7
2018	32.5	18.9	72.4	70.7	33	19.6	70.7	66.8
**VII**								
2016	32.3	19.4	215.9	72.6	33.3	20.3	134.2	69.5
2017	32.7	19.1	50.4	71.3	33.7	20.1	51.1	65.7
2018	30.5	19.9	142.3	72.5	31.3	20.5	130.4	69.8
**VIII**								
2016	30.3	17.9	47.7	75.3	31.6	18.7	71.6	71.5
2017	35.3	19.9	70.5	70.3	36.7	20.7	89.4	68.7
2018	32.8	20.6	70.9	73.5	33.7	20.9	97.6	72.4
**IX**								
2016	28.4	15.5	18.9	79.6	30	16.1	27.8	78.5
2017	26.9	13.5	172.1	82.7	29	14	179.4	82
2018	29.2	15.6	42.8	75.8	30	15.9	72.7	75.5
**X**								
2016	23.8	7.8	117.3	87.4	25	8.4	117.5	84.9
2017	23.4	9.7	78	84.4	24.9	10.2	80.8	84
2018	23.7	10.4	52.3	79.6	24.7	10.8	47.8	80.3

**XI**								
2016	19	3.7	33.4	86.9	18.4	4.2	42.4	83.9
2017	15.8	4.1	56.6	86.5	17	5	58.3	84.9
2018	21.9	4.5	11.7	87.5	22	5	8.8	85
**XII**								
2016	10.7	0.2	35.5	88	11.6	0.9	27	82.1
2017	14.5	1.7	23.4	82.4	14.8	2.6	23.4	79.8
2018	9.3	1.2	51.2	89.9	10.5	1.8	47	86.6

Table S3. Three-year average values of the selected basic climatic parameters measured at two different meteorological stations in the study area (IMGW code: 250190390 and 350190566) between January 2016 and December 2018. Data source: The Institute of Meteorology and Water Management - National Research Institute 2023.

	**Parameter**			
**Weather station**	**Three-year average maximum temperature [°C]**	**Three-year average monthly temperature [°C]**	**Three-year average monthly precipitation [mm]**	**Three-year average relative humidity [%]**
**Kraków-Obserwatorium**	24.16	10.23	59.37	75.06
**Kraków-Balice**	23.09	9.50	56.01	78.36
